# Mitochondrial retrograde signaling connects respiratory capacity to thermogenic gene expression

**DOI:** 10.1038/s41598-017-01879-x

**Published:** 2017-05-17

**Authors:** Minwoo Nam, Thomas E. Akie, Masato Sanosaka, Siobhan M. Craige, Shashi Kant, John F. Keaney Jr, Marcus P. Cooper

**Affiliations:** Division of Cardiovascular Medicine, Department of Medicine, University of Massachusetts Medical School, Worcester, MA 01605 USA

## Abstract

Mitochondrial respiration plays a crucial role in determining the metabolic state of brown adipose tissue (BAT), due to its direct roles in thermogenesis, as well as through additional mechanisms. Here, we show that respiration-dependent retrograde signaling from mitochondria to nucleus contributes to genetic and metabolic reprogramming of BAT. In mouse BAT, ablation of LRPPRC (LRP130), a potent regulator of mitochondrial transcription and respiratory capacity, triggers down-regulation of thermogenic genes, promoting a storage phenotype in BAT. This retrograde regulation functions by inhibiting the recruitment of PPARγ to the regulatory elements of thermogenic genes. Reducing cytosolic Ca^2+^ reverses the attenuation of thermogenic genes in brown adipocytes with impaired respiratory capacity, while induction of cytosolic Ca^2+^ is sufficient to attenuate thermogenic gene expression, indicating that cytosolic Ca^2+^ mediates mitochondria-nucleus crosstalk. Our findings suggest respiratory capacity governs thermogenic gene expression and BAT function via mitochondria-nucleus communication, which in turn leads to either a thermogenic or storage mode.

## Introduction

Brown adipose tissue (BAT) generates heat to combat cold stress^1^. When activated by cold or β-agonists, BAT oxidizes glucose and lipids (in the form of fatty acids) to fuel uncoupling protein 1 (UCP1)-mediated uncoupled respiration, which drives non-shivering thermogenesis^2^. Due to this unique energy-burning property, BAT has the potential to mitigate obesity^3, 4^. Since functional BAT exists in adult human subjects^1^ and likewise has the potential to mitigate obesity, there is great interest in understanding the molecular and cellular pathways that dictate its development, recruitment and maintenance.

Thermogenesis from BAT relies on abundant mitochondria in the tissue^2^. This makes BAT capable of higher levels of respiration than any other tissues^5^. It is thus readily anticipated that the metabolic state of BAT is influenced by mitochondrial respiration. Any circumstance where respiratory activity is low leads to reduced substrate oxidation, and will drive lipid accumulation in brown adipocytes like white adipocytes specialized for storing excess energy as lipids. On the other hand, high respiratory activity entails elevated substrate oxidation, thereby resulting in an energy-burning state in which stored lipids as well as uptaken glucose and lipids are oxidized. These two metabolic fates of BAT are also supported by two distinct gene programs. Lipogenic genes are enriched in both white and brown adipocytes, controlling fatty acid synthesis and esterification of glycerol with fatty acids. Thermogenic genes are uniquely expressed in BAT and oxidative genes are also highly present to enable high rates of fuel oxidation and respiration required for thermogenesis.

Previous studies have suggested that BAT senses its respiratory capacity and coordinates the expression of thermogenic genes to determine which metabolic states BAT adopts. Mice deficient for COX7RP, a factor that ensures proper function of mitochondrial respiratory complexes, exhibit increased lipid deposition in BAT^6^. Interestingly, expression of several thermogenic genes including *Ucp1*, *Dio2* and *Elovl3* is concurrently decreased. This suggests that mitochondria with impaired respiratory capacity communicate with the nucleus to attenuate expression of certain thermogenic genes. A similar mitochondria-nucleus communication has been described in brown adipocytes deficient for LRPPRC^7^. *Lrpprc* is the causative gene of the French-Canadian type of Leigh Syndrome, a rare metabolic and neurological disorder^8^. LRPPRC is a mitochondrial protein and has been shown to regulate mitochondrial-encoded electron transport chain (ETC) subunits and thus respiratory capacity by our laboratory and others^9–12^. LRPPRC knockdown causes a reduction in mitochondrial respiratory capacity and decreased expression of thermogenic genes including *Ucp1* and *Cidea* ^7^. However, gene expression profiling in BAT with impaired respiratory capacity is incomplete and the molecular mechanism by which mitochondria exert transcriptional control over those nuclear genes remains to be addressed.

In the present study, we explored the role of respiratory capacity in thermogenic gene expression by manipulating LRPPRC in an adipose-specific manner and by treating brown adipocytes with an inhibitor of mitochondrial respiration. We find that impaired respiratory capacity triggers a retrograde signaling pathway that represses thermogenic and oxidative genes, favoring decreased fuel oxidation and energy storage. Furthermore, we provide evidence that this information is transmitted via Ca^2+^-mediated mitochondrial retrograde signaling, which ultimately controls whether BAT participates in thermogenesis or energy storage.

## Results

### LRPPRC fat-specific knockout (FKO) mice exhibit impaired respiratory capacity in BAT

**Figure 1:**
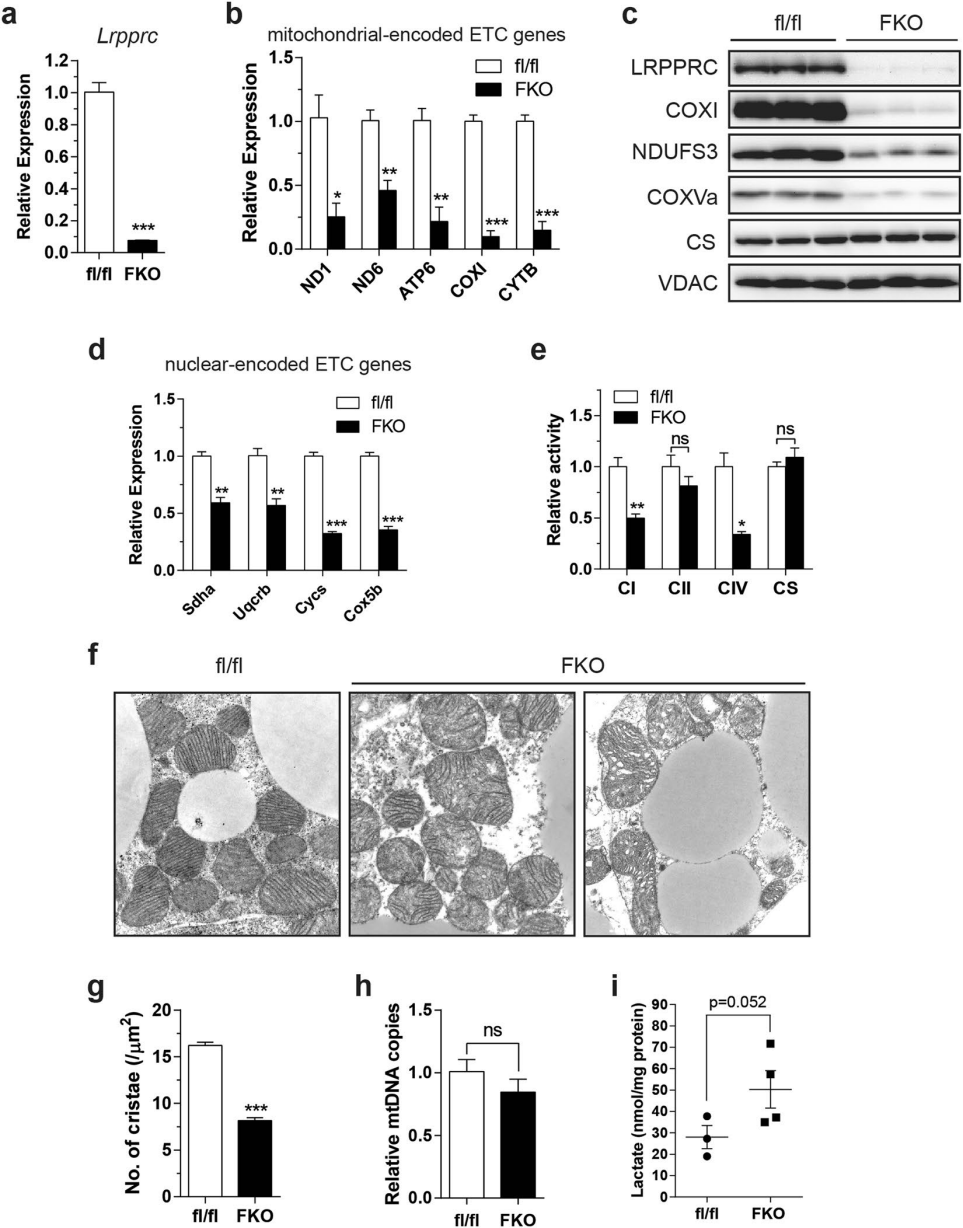
Respiratory capacity is impaired in BAT from LRPPRC fat-specific knockout (FKO) mice. (**a**,**b**) mRNA levels of *Lrpprc* (**a**) and mitochondrial-encoded ETC genes (**b**) in BAT from WT mice (fl/fl) and FKO mice. (**c**) Immunoblot of LRPPRC, COXI, NDUFS3, COXVa, VDAC, citrate synthase (CS) in BAT. (**d**) mRNA levels of nuclear-encoded ETC genes in BAT. (**e**) Complex activity in BAT. (**f**) TEM images of mitochondria in BAT (16,500X). (**g**) Number of cristae per μm^2^ of mitochondrion. 6–10 fields per mouse were analyzed (n = 3; total 174 mitochondria for fl/fl and 120 for FKO). (**h**) mtDNA content in BAT. (**i**) Lactate levels in BAT. (**a**–**e**,**h**) 11–12 week-old male, n = 3–5. (**f**,**g**) 14 week-old male, n = 3. Data are mean ± SEM. *P < 0.05, **P < 0.01, ***P < 0.001, one-tailed (**i**) and two-tailed unpaired Student’s *t-*test (**a**,**b**,**d**,**e**,**g**,**h**). CS, citrate synthase; mtDNA, mitochondrial DNA.

To examine whether respiratory capacity controls BAT gene expression *in vivo*, we generated fat-specific LRPPRC knockout mice (hereafter, FKO mice) by crossing LRPPRC floxed mice with Adiponectin-Cre mice. mRNA and protein levels of LRPPRC was reduced by >90% in BAT from FKO mice (Fig. 1a,c). Compared to WT mice (LRPPRC fl/fl), expression of mitochondrial-encoded ETC genes were globally reduced and COXI protein levels were also decreased (Fig. 1b,c). Interestingly, several nuclear-encoded subunits were also reduced at both mRNA and protein level (Fig. 1c,d). Abrogated expression of the ETC subunits resulted in impaired activities of respiratory complexes (Fig. 1e). Electron microscopy and image analysis revealed that WT mitochondria exhibited tightly packed lamellar cristae whereas FKO mitochondria displayed dysmorphic cristae architecture alongside reduced number of cristae (Fig. 1f,g). This is in agreement with the previous observation that heart-specific loss of LRPPRC leads to disorganized cristae^13^. Deficits in respiratory capacity were unlikely due to large changes in mitochondrial biogenesis, since markers of mitochondrial mass (VDAC, citrate synthase and mtDNA) were unchanged (Fig. 1c,h). Furthermore, lactate levels were increased 1.8-fold in FKO mice (Fig. 1i), consistent with previous studies demonstrating that pharmacological inhibition of the ETC causes increased lactate production due to increased glycolysis^14^.

### Impaired respiratory capacity attenuates thermogenic gene expression

**Figure 2:**
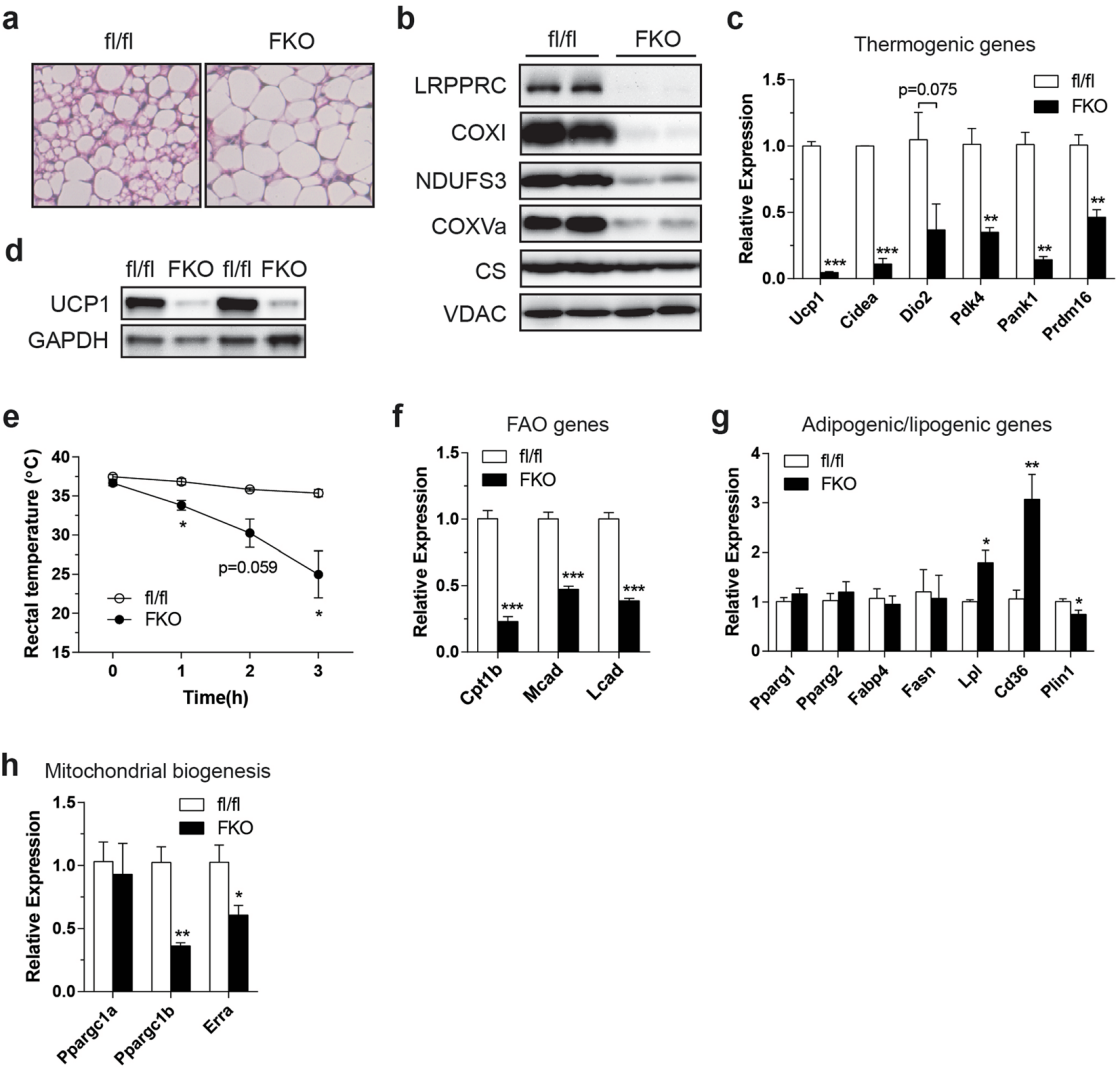
Impaired respiratory capacity attenuates thermogenic and oxidative gene expression in BAT from LRPPRC FKO mice living at 30 °C. (**a**) H&E staining of BAT. (**b**) Immunoblot of LRPPRC, COXI, NDUFS3, COXVa, CS and VDAC in BAT. (**c**) mRNA levels of thermogenic genes in BAT. (**d**) Immunoblot of UCP1 and GAPDH (loading control) in BAT. (**e**) Core temperature of control and LRPPRC FKO mice during acute cold exposure at 4 °C. (**f–h**) mRNA levels of FAO genes (**f**), adipogenic/lipogenic genes (**g**) and mitochondrial biogenesis genes (**h**) in BAT. 12–14 week-old male were used, n = 3–4. Data are mean ± SEM. *P < 0.05, **P < 0.01, ***P < 0.001, two-tailed unpaired Student’s *t-*test (**c**,**e**–**h**).

Having established a model of deficient respiratory capacity in BAT, we assessed BAT function and gene expression. On gross examination, BAT from FKO mice housed at room temperature (22 °C at our facility) was pale and enlarged (Supplementary Fig. 1a, upper). Increased lipid deposition with unilocular droplets was apparent in histological sections, an appearance associated with reduced respiratory activity (Supplementary Fig. 1a, lower). Although *Ucp1* mRNA levels were decreased, we observed that UCP1 protein was stabilized in FKO mice housed at room temperature (Supplementary Fig. 1b,c). 22 °C is a mild cold stressor to mice and such stabilization of UCP1 protein in cooler environments has been reported^15^. Upon acute cold exposure, these mice were not cold sensitive in spite of impaired respiratory capacity (Supplementary Fig. 1d). Although not formally assessed, augmented shivering thermogenesis due to housing under mild cold stress may compensate for UCP1-mediated non-shivering thermogenesis, enabling effective defense against cold. Cold also stimulates β-adrenergic signaling^3^. Since β-adrenergic signaling is a key regulator of both thermogenic and respiratory programs^16, 17^, we sought to determine whether impaired respiratory capacity *per se* affects BAT function and gene expression under circumstances devoid of β-adrenergic stimulation. To do so, mice were acclimated at thermoneutrality (30 °C) for 4 weeks, a timeframe that is sufficient to offset the impacts of thermal stress. Even at thermoneutrality, FKO mice maintained larger lipid droplets in BAT (Fig. 2a). Like FKO mice housed at room temperature, thermoneutral-acclimated FKO mice displayed robust depletion of LRPPRC and severe reduction in levels of COXI and nuclear-encoded respiratory subunits while VDAC was unchanged and CS was slightly reduced (Fig. 2b). In these mice, expression of thermogenic genes was severely decreased (Fig. 2c). Notably, both *Ucp1* mRNA and protein levels were severely reduced (Fig. 2c,d), and mice were exquisitely sensitive to cold stress (Fig. 2e).

We next assessed expression of genes that regulate fatty acid oxidation (FAO), adipogenesis, lipogenesis and mitochondrial biogenesis. Interestingly, FAO genes were globally reduced (Fig. 2f). Alongside decreased nuclear-encoded ETC genes, down-regulation of the FAO genes may favor transitioning of BAT into an energy-storing mode. In contrast, *Pparg*, a master regulator of adipogenesis, and its target lipogenic genes were unaltered or upregulated (Fig. 2g). *Ppargc1b* and *Erra* (*Esrra*) mRNA levels were reduced but not *Ppargc1a* mRNA (Fig. 2h). Although these genes are involved in mitochondrial biogenesis, as stated earlier, markers of mitochondrial mass were unchanged, suggesting alterations in various gene programs were not simply the result of reduced mitochondrial biogenesis. Mice housed at room temperature showed almost identical expression patterns of the aforementioned genes (Supplementary Fig. 1b,e,f,g), suggesting that mitochondrial retrograde signaling acts independent of β-adrenergic signaling. Furthermore, in support of normal β-adrenergic signaling in FKO mice living at thermoneutrality, the relative fold change for induction of *Ppargc1a* and *Ucp1* was comparable to control mice, following a cold stress (Supplementary Fig. 2a,b). If some brown adipocytes still contained residual LRPPRC, possibly due to inefficient recombination, one would predict a normal fold change of gene induction, following cold exposure. To exclude this possibility, we measured phosphorylated PKA, which is activated by β-adrenergic signaling. In BAT, pPKA was unchanged in FKO mice living at room temperature, further supporting that the β-adrenergic signaling pathway was not altered (Supplementary Fig. 2c,d).

In summary, these data indicate that impaired respiratory capacity triggers a retrograde signaling pathway that represses thermogenic and oxidative genes, favoring decreased fuel oxidation and thus energy storage. This may explain why lipid accumulation was increased in LRPPRC-deficient BAT.

### Impaired respiratory capacity interferes with the recruitment of PPARγ to thermogenic gene promoters

**Figure 3:**
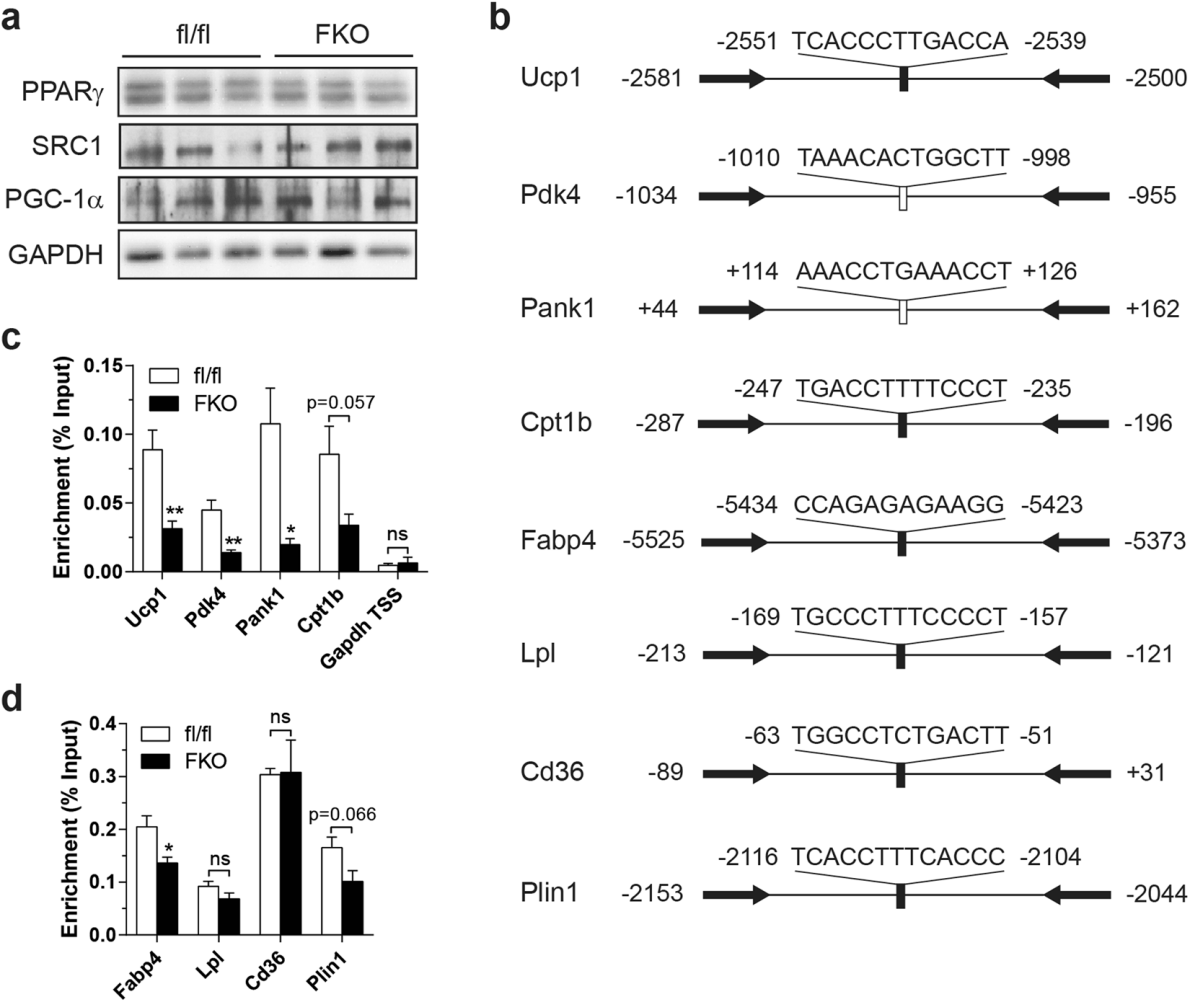
Impaired respiratory capacity influences the recruitment of PPARγ. (**a**) Immunoblot of PPARγ, SRC1, PGC-1α and GAPDH (loading control) in BAT. (**b**) Schematic diagram depicting the positions of primers used for ChIP and the positions/sequences of PPREs of the genes assessed by ChIP assays. Filled bar: previously identified PPRE; Open bar: putative PPRE. The arrows indicate the positions of primers. (**c**,**d**) ChIP analysis of thermogenic and oxidative gene enhancer/promoters (**c**) and lipogenic gene promoters (**d**) using PPARγ antibody in BAT. 12–14 week-old male at 30 °C. (**c**,**d**) n = 4–5. Data are mean ± SEM. *P < 0.05, **P < 0.01, two-tailed unpaired Student’s *t-*test (**c**,**d**).

We were interested in the transcriptional basis by which deficits in respiratory capacity affects thermogenic gene expression. PPARγ governs many aspects of brown fat development and maintenance^18, 19^. Protein levels of PPARγ and coactivators including SRC1 and PGC-1α, however, were unchanged in FKO mice (Fig. 3a). Even so, PPARγ has been shown to exhibit promoter specificity under certain metabolic conditions^20^. We, therefore, queried whether the recruitment of PPARγ to various transcriptional regulatory units was altered using ChIP assays (Fig. 3b). As shown in Fig. 3c, the recruitment of PPARγ to the enhancer region of *Ucp1* and the promoters of other thermogenic genes was reduced in FKO mice. Because PPARγ is required for the expression of these genes^21, 22^, reduced recruitment to these regulatory regions might explain their reduced transcription. However, recruitment of PPARγ to the promoters of lipogenic genes was unchanged with some minimally affected in FKO mice (Fig. 3d), a finding consistent with intact lipogenic gene expression (Fig. 2g). These data suggest that mitochondrial retrograde signaling influences promoter specific recruitment of PPARγ, a metabolic switch that governs whether or not BAT adopts a thermogenic or storage phenotype.

### Cytosolic calcium may mediate retrograde signals from mitochondria to nucleus

**Figure 4:**
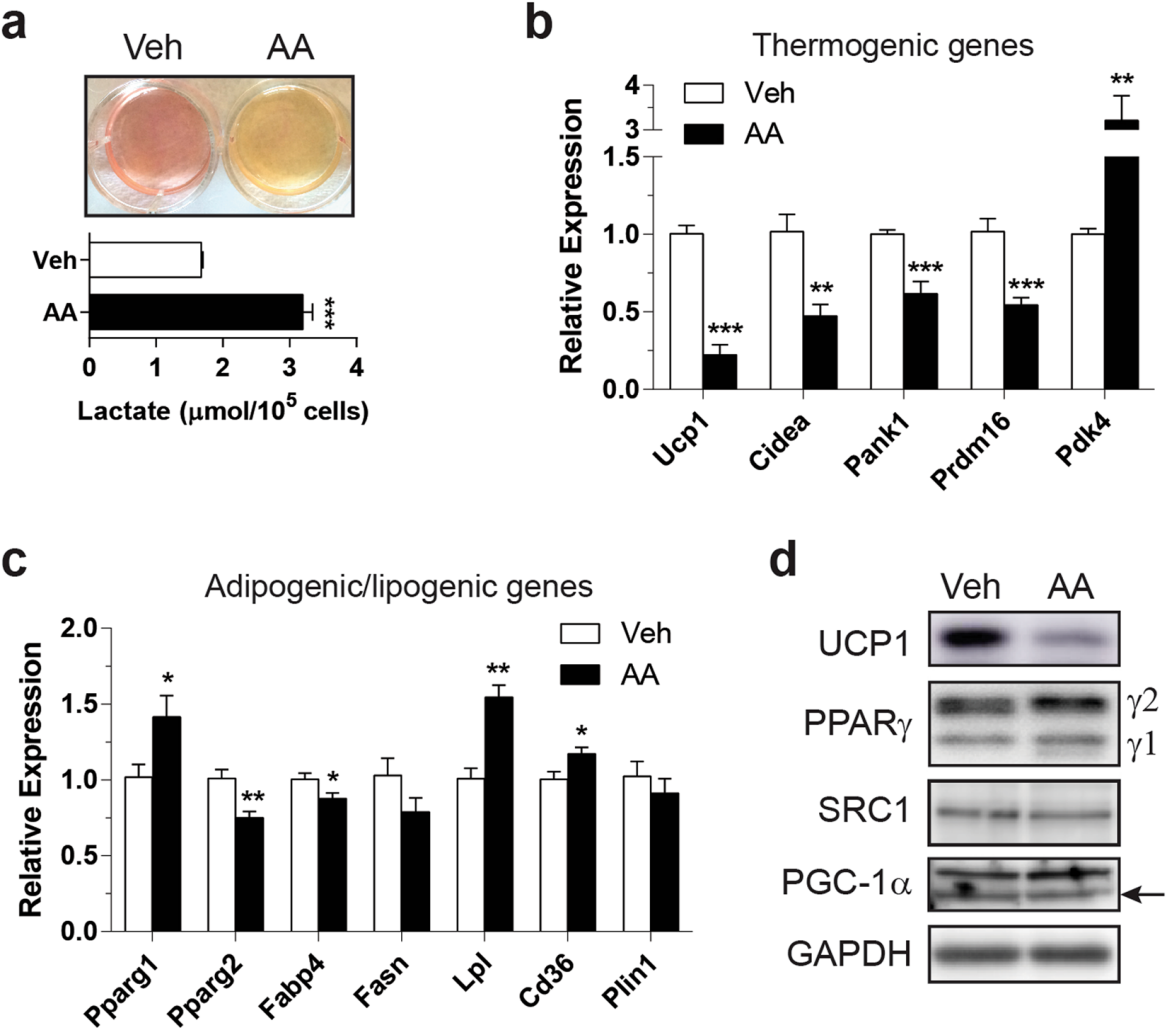
Pharmacological inhibition of respiratory complex mimics LRPPRC ablation in cultured brown adipocytes. (**a**) Representative image of acidic media (upper) and lactate production (lower) in primary brown adipoytes treated with 10 nM AA for 18 hr. (**b**,**c**) mRNA levels of thermogenic genes (**b**) and adipogenic/lipogenic genes (**c**). (**d**) Immunoblot of UCP1, PPARγ, SRC-1, PGC-1α and GAPDH (loading control). Data are mean ± SEM. *P < 0.05, **P < 0.01, ***P < 0.001, two-tailed unpaired Student’s *t*-test (**a**–**c**).

To further identify the mechanism by which thermogenic gene expression is attenuated, we established two different cell culture models with impaired respiratory capacity: genetic impairment of respiratory capacity via LRPPRC knockdown and pharmacological inhibition of respiratory complex. Similar to our previous observation^7^ and FKO mice, brown adipocytes with LRPPRC knockdown exhibited decreases in ETC subunits and UCP1 (Supplementary Fig. 3). As a pharmacological model, we treated brown adipocytes with Antimycin A (AA), an inhibitor of complex III. Since electrons entered from complexes I and II converge at complex III, inhibiting complex III will block the entire electron transit, which impairs respiratory capacity. As in BAT from FKO mice, lactate levels were increased in AA-treated brown adipocytes (Fig. 4a). Notably, AA treatment resulted in reduced mRNA levels of most of the thermogenic genes tested (Fig. 4b) but a minimal effect on lipogenic genes with some genes upregulated (Fig. 4c). UCP1 protein was also reduced whereas PPARγ and coactivators SRC-1 and PGC-1α were unaltered (Fig. 4d). Overall, both genetic and pharmacological impairment of ETC in cell culture recapitulates the findings from FKO mice, providing *in vitro* models to study downstream signaling pathway. These data also suggest that the effects of impaired respiratory capacity on thermogenic gene expression are cell autonomous.

**Figure 5:**
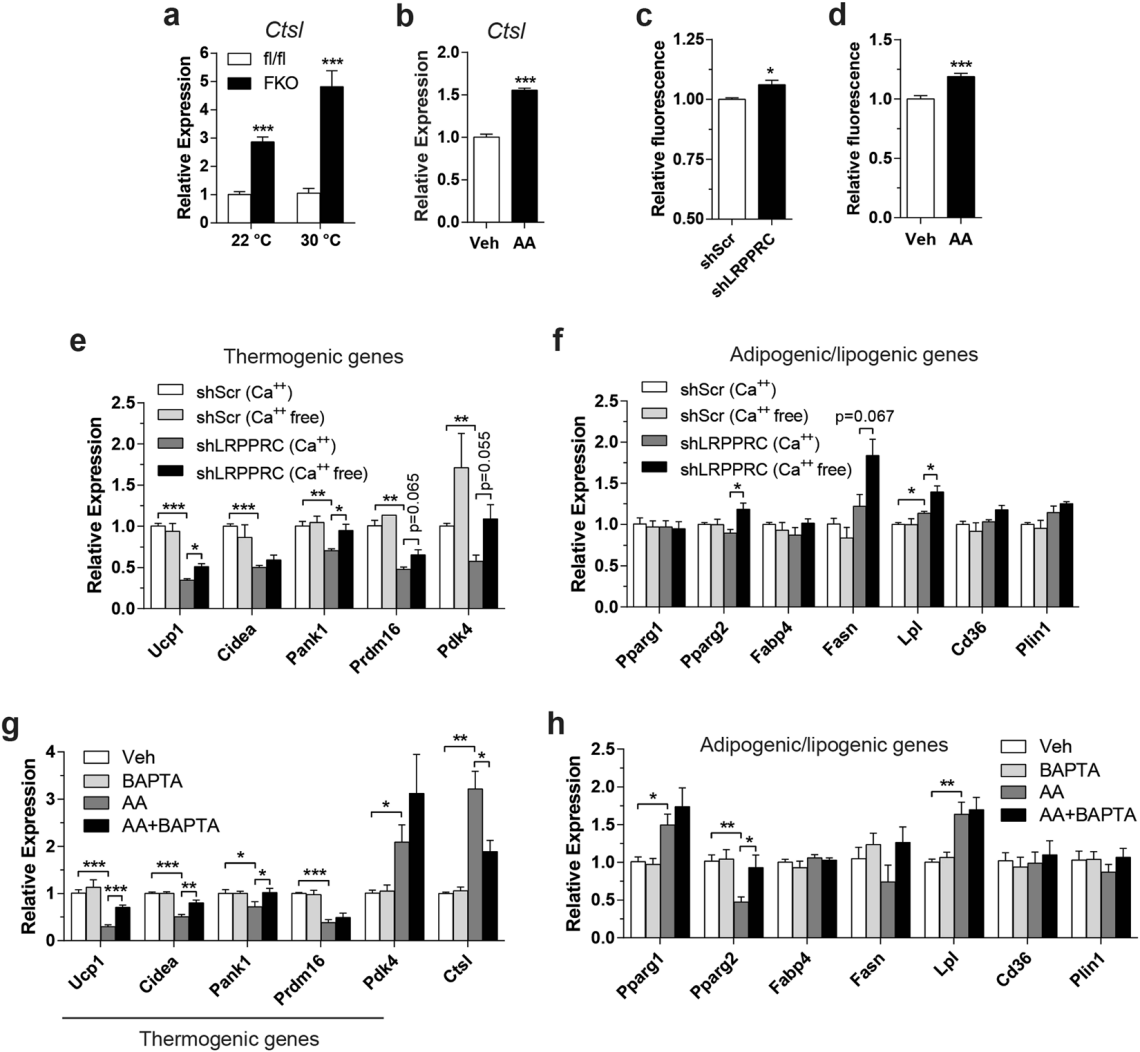
Cytosolic Ca^2+^ may serve as a signaling mediator between mitochondria and nucleus. (**a**,**b**) mRNA levels of *Ctsl* in BAT (**a**) and primary brown adipocytes (**b**). (**c**,**d**) Measurement of cytosolic Ca^2+^ in immortalized brown adipocytes with LRPPRC knockdown (**c**) or treated with AA (**d**) using a cell-permeable Ca^2+^-specific fluorescent indicator Fluo4-AM. (**e**,**f**) mRNA levels of thermogenic genes (**e**) and adipogenic/lipogenic genes (**f**) in LRPPRC knockdown cells. Cells were incubated in Ca^2+^-free media supplemented with 100 μM EGTA for 8–12 hr. (**g**,**h**) mRNA levels of thermogenic genes (**g**) and adipogenic/lipogenic genes (**h**) in immortalized brown adipocytes co-treated with AA and BAPTA. 40 μM BAPTA-AM (a cell-permeable form of BAPTA) was loaded into cells for 1 hr, followed by treatment with 20 nM AA for 18 hr. (**a**) n = 3–5. Data are mean ± SEM. *P < 0.05, **P < 0.01, ***P < 0.001, two-tailed unpaired Student’s *t-*test (**a**–**h**).

Several studies have shown that ETC dysfunction leads to increased cytosolic Ca^2+^ levels^23–25^. These studies demonstrated that Cathepsin L (*Ctsl*) was induced in a Ca^2+^-dependent manner. First, as an indirect measure of altered cytosolic Ca^2+^ levels, we quantified *Ctsl* mRNA in BAT from LRPPRC FKO mice and AA-treated cells. *Ctsl* gene expression was induced in FKO mice housed at 22 °C and 30 °C (Fig. 5a) and AA-treated cells (Fig. 5b), indicating elevated levels of cytosolic Ca^2+^. Next, we quantified steady-state levels of cytosolic Ca^2+^, and observed significant increases in LRPPRC knockdown cells (Fig. 5c) and AA-treated cells (Fig. 5d). We then examined whether reduction of free cytosolic Ca^2+^ levels can rescue the repression of thermogenic genes. Ca^2+^-free media was able to rescue reduced thermogenic gene expression in LRPPRC knockdown cells, the effect being partial for some genes (Fig. 5e). Ca^2+^-free media had no substantial impact on lipogenic genes (Fig. 5f). In addition, with minimal effects on lipogenic genes, BAPTA-AM, a cell-permeable form of Ca^2+^ chelator BAPTA, partially rescued decreases in thermogenic genes (Fig. 5g,h), suggesting that multiple mediators are involved in thermogenic gene regulation in both models. BAPTA was also able to partially reverse AA-dependent induction of *Ctsl* (Fig. 5g), indicating that BAPTA effectively blocked Ca^2+^-dependent alterations in gene expression. In summary, under impaired respiratory capacity, mitochondrial-nuclear crosstalk is likely multi-factorial relying in part on cytosolic Ca^2+^.

**Figure 6:**
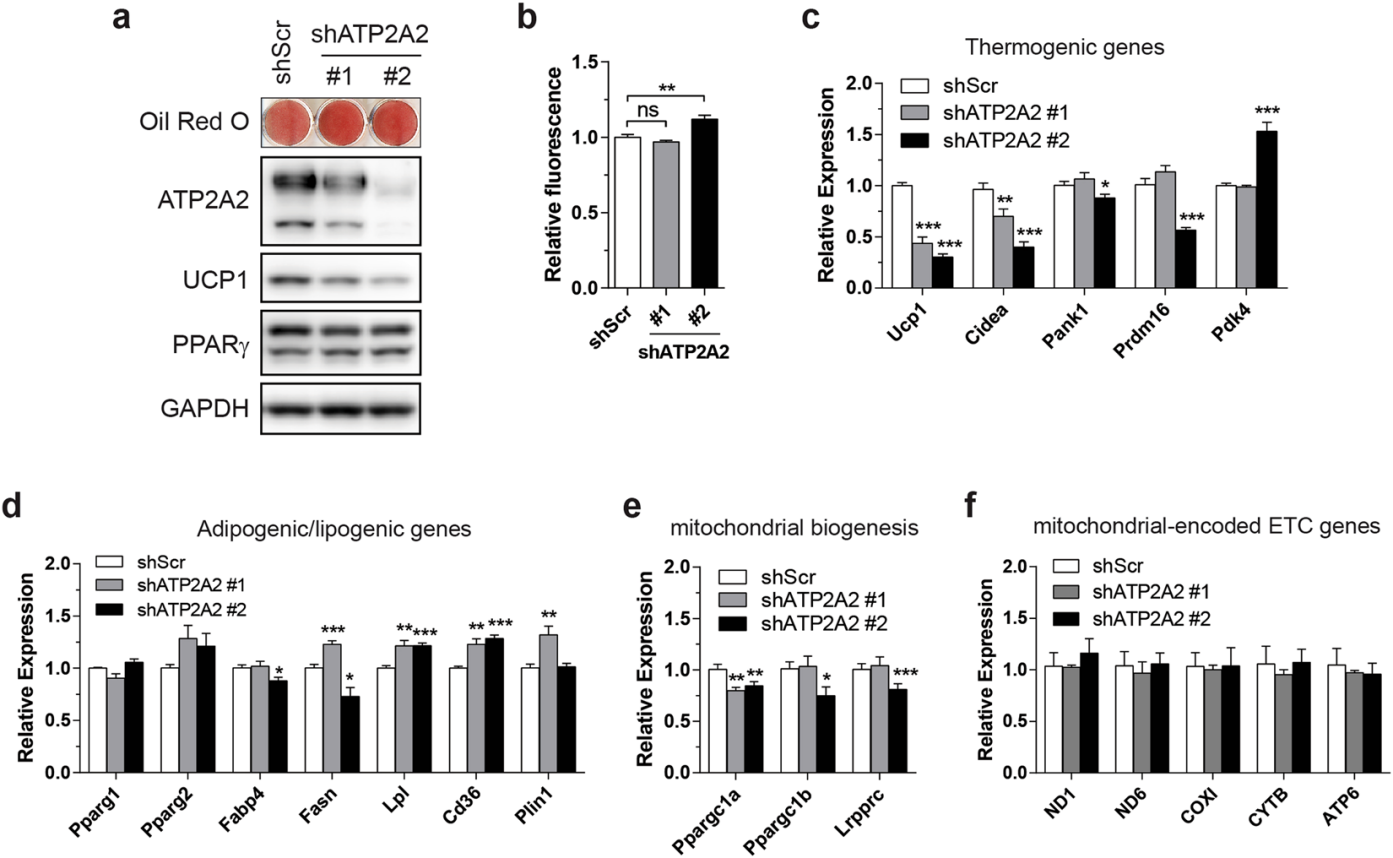
Increasing cytosolic Ca^2+^ is sufficient to attenuate thermogenic gene expression. (**a**) Oil red O staining of immortalized primary brown adipocytes stably transduced with ATP2A2 shRNA and immunoblot of ATP2A2, UCP1, PPARγ and GAPDH (loading control). (**b**) Measurement of cytosolic Ca^2+^ in immortalized brown adipocytes stably expressing shATP2A2. (**c–f**) mRNA levels of thermogenic genes (**c**), adipogenic/lipogenic genes (**d**), mitochondrial biogenesis genes (**e**) and mitochondrial-encoded ETC genes (**f**). Data are mean ± SEM. *P < 0.05, **P < 0.01, ***P < 0.001, two-tailed unpaired Student’s *t-*test (**b**–**f**).

Finally, we tested whether increasing cytosolic Ca^2+^ mimics the effects of impaired respiratory capacity to some extent by silencing sarco/endoplasmic reticulum (SR/ER) Ca^2+^-ATPase (SERCA) in brown adipocytes. Since SERCA transports Ca^2+^ from cytosol into SR/ER at the expense of ATP, cytosolic Ca^2+^ is expected to be increased in SERCA-deficient cells. Although three paralogous genes encode SERCA (*Atp2a1*, *Atp2a2* and *Atp2a3*), mouse brown adipocytes only express *Atp2a2* and *Atp2a3* with the latter being induced upon differentiation (data not shown). Immunoblot analysis confirmed protein expression of ATP2A2 (SERCA2) in cultured brown adipocytes but ATP2A3 (SERCA3) was undetectable with our immunoblotting methods in the same cells (Supplementary Fig. 4). Even if low levels of ATP2A3 protein are expressed, ATP2A3 has unusually low Ca^2+^ affinity, rendering it essentially inactive at normal intracellular Ca^2+^ concentration (≤0.1 μM)^26^. Therefore, we chose to silence a single isoform: ATP2A2. Two different sequences of ATP2A2 shRNA yielded moderate to severe silencing (Fig. 6a). Oil red O staining and unaltered PPARγ protein indicated no apparent effect of ATP2A2 knockdown on differentiation (Fig. 6a). Cytosolic Ca^2+^ was increased in cells with severe knockdown but not with modest knockdown (Fig. 6b); we speculate that any change that may be caused by moderate knockdown of ATP2A2 appears to be outside the detection range of the method. Alternatively, although this work focused on Ca^2+^ concentration, Ca^2+^ signaling is also transduced via cytosolic Ca^2+^ oscillations^27^. In cells with ATP2A2 (SERCA2) haploinsufficiency, there were no significant changes in baseline cytosolic Ca^2+^ levels^28^. Instead, the Ca^2+^ oscillatory pattern was altered^28^. This may explain why modest knockdown of ATP2A2 still had an impact on thermogenic genes, and the potential changes in Ca^2+^ oscillation may be another important factor in LRPPRC-deficient cells, AA-treated cells and cells with severe knockdown of ATP2A2 along with increased cytosolic Ca^2+^ levels. Nonetheless, *Ucp1* mRNA and protein were reduced in proportion to the extent of ATP2A2 knockdown (Fig. 6a,c). Levels of other thermogenic genes were also similarly decreased (Fig. 6c) whereas lipogenic genes were marginally affected (Fig. 6d) and genes regulating mitochondrial biogenesis exhibited minimal changes (Fig. 6e). Finally, *Lrpprc* and mitochondrial-encoded respiratory genes were unaltered, indicating that SERCA knockdown affects Ca^2+^ trafficking through a distinct mechanism (Fig. 6e,f). Together, these data support that cytosolic Ca^2+^ is one of the second messengers for mitochondrial retrograde signaling in brown adipocytes.

## Discussion

In this study, we tested whether reducing respiratory capacity in mouse BAT affects thermogenic gene expression and BAT function. We modeled impaired respiratory capacity by ablating LRPPRC in an adipose-specific manner. Impaired respiratory capacity activated retrograde signaling pathway to attenuate thermogenic and oxidative gene expression. The transcriptional basis for this repression was the reduced recruitment of PPARγ to the promoters of those genes. Using shRNA against LRPPRC, an inhibitor of respiratory complex and shRNA against SERCA pump in cultured brown adipocytes, and conversely the means of reducing cytosolic Ca^2+^ under respiration-impaired conditions, we also showed that Ca^2+^ mediates the crosstalk between mitochondria and nucleus. Overall, our work illustrates an adaptive coordination of respiratory capacity with the expression of BAT-enriched thermogenic genes.

Mitochondrial retrograde signaling is triggered by various mitochondrial stresses^29–31^. This signaling pathway affects nuclear gene expression, resulting in a multitude of cellular adaptive responses^29–31^. Our data highlight an adaptive response of brown adipocytes to impaired respiratory capacity, which is an unfavorable condition for thermogenesis. In contrast, adipose-specific loss of TFAM, an activator of mitochondrial transcription and positive regulator of mtDNA replication, had no such defects in BAT^32, 33^. Despite the reduced expression of mitochondrial-encoded ETC genes, however, oxygen consumption, FAO, and citrate synthase activity were paradoxically increased in TFAM-deficient BAT^32, 33^, which confounds interpretation. Importantly, the phenotypic similarities between LRPPRC ablation and pharmacological inhibition of respiratory complex exclude pleiotropic effects of LRPPRC loss. Moreover, impaired respiratory capacity was associated with remodeling of oxidative program in BAT. In LRPPRC FKO mice, gene programs involved in mitochondrial respiration (nuclear-encoded) and fatty acid oxidation were impaired. Some of the changes might be explained by reduced expression of *Ppargc1b* and *Erra* (*Esrra*), both of which govern mitochondrial biogenesis and fatty acid oxidation and ref. 17. It is noteworthy that LRPPRC depletion in hepatocytes had no overt effect on the oxidative gene program^34^. This differential gene regulation suggests tissue specificity of mitochondrial retrograde signaling. Attenuated expression of genes involved in fatty acid oxidation was not simply due to decreased mitochondrial content. Interestingly, impaired respiratory capacity in BAT was not associated with a compensatory increase in mitochondrial content. This is in contrast to inhibition of oxidative phosphorylation (OXPHOS) in skeletal muscle where induction of PGC-1 coactivators promotes mitochondrial biogenesis to presumably compensate for OXPHOS deficits^35^. This dichotomy is interesting as it indicates the status of respiratory capacity in BAT globally determines adipocyte function: storage versus heat dissipation. With normal respiratory capacity, BAT is committed to function as a thermogenic organ and thermogenic and oxidative gene expression is maintained. However, upon impaired respiratory capacity, a condition unfavorable for thermogenesis, thermogenic and oxidative gene expression is suppressed. Concurrently, glycolysis is increased, which can supplement ATP and enhance *de novo* lipogenesis^36^. These mechanisms converge to reprogram BAT into a storage mode.

One could intuitively believe that a coordinated regulation of thermogenic and lipogenic programs is required to ensure continuous fuel supply for thermogenesis in BAT. Indeed, cold promotes lipogenic function of BAT by inducing certain lipogenic genes and enhancing lipogenic flux to replenish lipid pools that are rapidly consumed during thermogenesis^37, 38^. However, in our experimental conditions for mice and cultured brown adipocytes, conditions that involve mild cold stress or no apparent cold challenge, we do not observe such coordinated regulation of thermogenic and lipogenic functions in BAT. Our work, therefore, suggests that respiratory capacity-Ca^2+^ axis is linked to ‘basal’ expression of thermogenic genes but not lipogenic genes. In addition, many thermogenic and lipogenic genes are controlled by PPARγ with the thermogenic genes generally coactivator-requiring but the lipogenic genes being coactivator-independent. The functions of PPARγ coactivators may be specifically impaired in BAT with impaired respiratory capacity, which could lead to independent regulation of thermogenic and lipogenic genes at least at basal state.

While we have shown that reduced recruitment of PPARγ to the promoters of thermogenic genes may be responsible for their attenuated expression, precisely how PPARγ is dislodged from those promoters remains unknown. As stated above, a coactivator complex consisting of PGC-1α, SRC-1/3 and other general coactivators is necessary for PPARγ-dependent thermogenic gene expression but is dispensable for expression of lipogenic genes^39, 40^. Based on our findings of attenuated (coactivator-dependent) thermogenic gene expression but intact (coactivator-independent) lipogenic gene expression, disrupted coactivator complex may be a potential mechanism. SRC-1 and SRC-3 are shown to be jointly required for recruitment of PPARγ to a PPRE site on the *Ucp1* enhancer in BAT but not to lipogenic gene promoters^39^. One possibility is that impaired respiratory capacity interferes with the function of SRC family as a PPARγ coactivator, leading to diminished docking of PPARγ on the thermogenic promoters. In addition, given a model in which binding of PGC-1α to PPARγ promotes recruitment of SRC-1 and CBP/p300^41^, abrogation of physical interaction between PPARγ and PGC-1α could indirectly hinder PPARγ docking by sequestering SRC-1 and possibly SRC-3 from PPARγ coactivator complexes.

We provide evidence of cytosolic Ca^2+^ as a signal that may mediate mitochondria-nucleus crosstalk triggered by impaired respiratory capacity in BAT. It has been reported that cytosolic Ca^2+^ was increased by certain mitochondrial stresses including depletion of mtDNA and inhibition of the respiratory chain in various cell types, which ultimately affected nuclear gene expression^23, 25, 42, 43^. Defective respiratory chain function is also associated with deranged mitochondrial Ca^2+^ handling^44, 45^. We speculate that impaired respiratory capacity impairs Ca^2+^ buffering by mitochondria, leading to increased cytosolic Ca^2+^. In brown adipocytes with genetically or pharmacologically impaired respiratory capacity, increased cytosolic Ca^2+^ was at least in part responsible for repressed thermogenic genes. To our knowledge, this is a first report describing Ca^2+^-mediated mitochondrial retrograde signaling in BAT. In contrast, Ca^2+^ is also known to positively regulate BAT thermogenesis. Unlike our model, β3-adrenergic stimulation of brown adipocytes led to a rise in intracellular Ca^2+^ that is evoked from mitochondria, ER and entry across plasma membrane^46^. It has been suggested that Ca^2+^ influx mediated by TRPV2, a Ca^2+^-permeable non-selective cation channel, was required for isoproterenol-induced expression of *Ppargc1a* and *Ucp1* in brown adipocytes^47^. Moreover, activation of TRPM8, a cold-sensing non-selective cation channel, induced UCP1 expression through Ca^2+^-mediated PKA phosphorylation in brown adipocytes^48^. This discrepancy suggests that mitochondrial retrograde signaling involves a Ca^2+^ signaling pathway that is distinct from the one in stimulated brown adipocytes. Furthermore, the sources of Ca^2+^ could be an important determinant of the responses in brown adipocytes. For example, neurons respond distinctly to different sources of Ca^2+^ influx^49^. This ‘source-specificity hypothesis’ may explain why the outcomes of Ca^2+^ signaling pathways activated by β3-adrenergic stimulation (Ca^2+^ from extracellular space, ER and mitochondria) and mitochondrial retrograde signaling (Ca^2+^ from mitochondria) are different. Although unclear at present, investigating how Ca^2+^ influences PPARγ and possibly its coactivator complex may help elucidate the distinct mechanism, which could prove of therapeutic utility.

In summary, our study demonstrates that BAT coordinates its respiratory status with the expression of thermogenic and oxidative genes through retrograde signaling to determine its metabolic commitment. When respiratory capacity is impaired, BAT adopts a storage phenotype by turning off thermogenic genes and down-regulating genes involved in fuel oxidation. Our work may provide the important framework for future research on mitochondrial control of thermogenic gene pathway and energy dissipation.

## Methods

### Animals

*Lrpprc* ^*flox/flox*^ mice were generated as previously described^50^. To ablate *Lrpprc* in a fat-specific manner, *Lrpprc* ^*flox/flox*^ mice were crossed to *Adipoq-Cre* mice. The resultant *Lrpprc* ^*flox/*+^;*Adipoq-cre/0* mice were then crossed to *Lrp130* ^*flox/flox*^ mice to produce *Lrpprc* ^*flox/flox*^; *Adipoq-cre/0* mice (termed LRPPRC FKO). Our final breeding strategy was to cross *Lrpprc* ^*flox/flox*^ mice to LRPPRC FKO mice, resulting in *Lrpprc* ^*flox/flox*^: LRPPRC FKO = 1:1. Because there was no metabolic or genetic differences between WT, *flox/flox* mice or *Adipoq-Cre/0* mice, comparisons between control (flox/flox) and *Lrpprc* ^*flox/flox*^; *Adipoq-cre/0* mice were used for this study. Mice were maintained in 12-hour light/12-hour dark cycle. *Lrpprc* ^*flox/flox*^ mice and *Adipoq-Cre* mice are on a C57BL6/J background. All animal experiments were performed in accordance with protocols approved by UMMS IACUC.

### Cold exposure

Mice were acclimatized at 30 °C for 4 weeks. The mice were then housed individually and acutely exposed to cold (4 °C). Rectal temperature was measured hourly using a digital thermometer (MicroTherma 2 T, Thermoworks) and a rectal probe (RET-3, Physitemp) for up to 8 hours. The end point was a 10 °C drop in temperature (approximately 27 °C) and mice were immediately euthanized.

### Histology

For hematoxylin and eosin (H&E) staining, brown adipose tissue was collected, washed in ice-cold PBS and fixed in 4% paraformaldehyde with gentle shaking at 4 °C overnight. Subsequent procedures were performed by UMass morphology core facility.

### Transmission electron microscopy (TEM)

BAT was dissected and chopped finely in PBS, followed by overnight fixation in 0.1 M cacodylate buffer (pH 7.2) containing 2.5 M glutaraldehyde. Sample preparation and image acquisition were performed by UMMS core electron microscopy facility using FEI Tecnai Spirit 12 TEM.

### Reverse transcription-quantitative PCR (RT-qPCR)

Total RNA was isolated from cell culture using Trizol according to the manufacturer instructions (Invitrogen). For mouse adipose tissue, the aqueous phase prepared from Trizol extraction was subject to acidic phenol extraction (pH 4.4) to remove residual lipid, followed by purification using RNeasy (Qiagen) or GeneJET RNA columns (Thermo Scientific). cDNA was synthesized from 0.5–1 μg RNA, using MultiScribe reverse transcriptase (Applied Biosystems). Quantitative PCR was performed using Fast SYBR Green Master Mix (Applied Biosystems) on a 7500 FAST Real-Time PCR system (Applied Biosystems). For a normalization purpose, several widely used internal control genes were tested in all experimental groups and the most stable one was selected. Relative gene expression was calculated by the comparative C_T_ method. Coefficient of variation for the reference genes was less than 1% across samples. Primers are listed in Table S1.

### Quantification of mitochondrial DNA content

Approximately 5–10 mg of frozen brown fat were lysed in 300 μL tissue lysis buffer (50 mM Tris-Cl pH 7.5, 50 mM EDTA pH 8.0, 100 mM NaCl, 1% Triton X-100, 5 mM DTT and 100 mg/ml proteinase K) at 56 °C for 6 hours. DNA isolation and quantitative PCR were performed as previously described^50^.

### Immunoblotting

Approximately 10 mg of frozen brown fat or 50 mg of frozen inguinal white fat were placed in ice-cold RIPA buffer supplemented with protease inhibitor cocktail (Sigma), phosphatase inhibitor cocktail (Sigma) and sodium β-glycerophosphate. The tissue was then homogenized using a bead mill homogenizer (Qiagen TissueLyzer). The lysates were vortexed vigorously for 5 seconds, incubated on ice for 10 minutes and cleared by centrifuging at 13200 rpm for 15 minutes at 4 °C. Preparation of lysates from cell culture was performed as above without using TissueLyser. Protein concentration was determined using a BCA kit (Pierce). Indicated amounts of proteins were separated on a polyacrylamide gel and blotted onto a PVDF membrane. The membrane was blocked in 5% non-fat milk in TBS-tween, followed by incubation with primary antibodies directed against proteins of interest and HRP-conjugated secondary antibodies. The protein signals were visualized with Amersham ECL kit (GE Healthcare) or WestPico ECL kit (Thermo Scientific) and digitally recorded using Amersham Imager 600 (GE Healthcare). The antibodies used are as follows: LRPPRC (produced in-house using mice); UCP1 (U6382, Sigma); PPARγ (sc-7273, Santa Cruz); COXI (ab14705, Abcam); COXVa (ab110262, Abcam); NDUFS3 (ab110246, Abcam); Citrate Synthase (GTX110624, GeneTex); VDAC (4866, Cell Signaling); GAPDH (sc-25778, Santa Cruz); ATP2A2 (sc-8095, Santa Cruz); ATP2A3 (sc-81759, Santa Cruz).

### Lactate measurement

Lactate levels were measured in homogenates prepared from approximately 5–10 mg of BAT using Lactate Assay kit II (Biovision). For AA-treated cells, lactate secretion was measured in culture medium using the same kit.

### Complex activity and citrate synthase activity

Complex activity and citrate synthase activity were measured in BAT homogenates as previously described^50, 51^.

### Chromatin immunoprecipitation (ChIP)

Interscapular brown fat was collected, washed with ice-cold PBS and finely minced. Minced tissue was cross-linked in 10 volume of PBS containing 1% paraformaldehyde for 10 minutes at room temperature on a rotator. Cross-linking was quenched by adding a final concentration of 125 mM glycine. The samples were then dounced on ice 10 times, washed twice with ice-cold PBS. Disaggregated tissue was placed in 1 ml of RSB buffer (3 mM MgCl2, 10 mM NaCl, 10 mM Tris-Cl pH 7.4, 0.1% NP-40 and protease inhibitor cocktail [Sigma]), dounced on ice 30 times, incubated on ice 5 minutes and filtered through 100 μM cell strainer. The homogenate was centrifuged and the pellet was resuspended in nuclei lysis buffer (1% SDS, 10 mM EDTA, 50 mM Tris-Cl pH 8.1 and protease inhibitor cocktail). The chromatin was subject to three sonication cycles (a cycle of 10 minutes with a duty of 30 seconds on/30 seconds off) using Diagenode Bioruptor. The samples were cleared by centrifugation, diluted in ChIP dilution buffer (1% Triton-X100, 2 mM EDTA, 150 mM NaCl, 20 mM Tric-Cl pH 8.0 and protease inhibitor cocktail) and incubated overnight at 4 °C with 2 μg of anti-PPARγ antibody (sc-7196, Santa Cruz). Immunocomplexes were recovered with protein A/G beads (Pierce) and eluted DNA was further purified using the QIAquick gel extraction kit (Qiagen). Quantitative real-time PCR was performed using specific primers for the indicated gene promoters. Primers are listed in Table S2.

### Cell culture

Primary stromal vascular fraction containing preadipocytes was isolated from interscapular depot of P0-P2 newborn Swiss Webster mice (Taconic Biosciences) as previously described^7^. Prior to induction of differentiation, primary brown preadipocytes were grown to >90% confluence in DMEM (Corning) containing 20% FBS, 20 mM HEPES and 1 mM sodium pyruvate. Immortalized brown preadipocytes were grown in the same condition except that 10% FBS was used. For adipocyte differentiation, confluent cells were exposed to DMEM containing 0.5 μM dexamethasone, 125 μM indomethacin, 0.5 mM isobutylmethylxanthine, 20 nM insulin, 1 nM T3 and 10% FBS for 2 days, after which medium was switched to DMEM containing 20 nM insulin, 1 nM T3 and 10% FBS, and replenished every 2 days. On day 6 post differentiation, cells were treated as indicated. At least three independent experiments were performed.

### Lentiviral transduction

shRNA oligomers were annealed and cloned into pLKO.1-hygro lentiviral vector as described in the protocol available from Addgene. 21 bp sense sequences for shLRPPRC and shATP2A2 are as follows: shLRPPRC: 5′-TGAAGCTAGATGACCTGTTTC-3′; shATP2A2 #1: 5′-GGCGAGAGTTTGATGAATTAA-3′; shATP2A2 #2: 5′-TGACTCTGCTTTGGATTATAA-3′; shScramble (shScr): negative control; Addgene #1864. To produce lentiviruses, HEK-293T cells were transfected with the pLKO.1-hygro construct, psPAX2 and pMD2.G using lipofectamine 2000 (Invitrogen) according to the manufacturer instructions. Medium was replaced after 16–20 hours of incubation with the DNA:lipofectamine mixture. At 48 hours post transfection, medium was harvested and passed through 0.45 μm filter (Thermo Scientific). The medium was diluted 2-fold in fresh medium and added to subconfluent immortalized brown preadipocytes plated in 12-well plate with 4 μg/ml polybrene. After overnight incubation, cells were replenished with fresh medium and incubated for additional 24–30 hours. Cells were then trypsinized and seeded in 100 mm dish in the presence of 400 μg/ml hygromycin, after which medium was replaced every 48 hours. At day 5 post selection, hygromycin was removed and cells were used for differentiation.

### Calcium measurement

Preadipocytes were plated and differentiated in a 96-well clear bottom black plate (Costar). Fully differentiated cells (day 6) were washed with 150 ul HBSS (Gibco 14175–095) supplemented with 1.8 mM CaCl_2_, 0.8 mM MgSO_4_, 1 nM T3 and 20 nM insulin. Cells were then loaded with 4 μM Fluo 4-AM (Invitrogen) in 100 ul HBSS for 1 hour at 37 °C (30 °C for LRPPRC knockdown cells and ATP2A2 knockdown cells), followed by two washes with 150 ul HBSS. Fluorescence was measured at 485/520 nm in 100 ul HBSS using a microplate reader (POLARstar Omega, BMG LABTECH). Three independent experiments were performed and each experiment included biological duplicates.

### Statistics

Statistical analyses were performed using GraphPad Prism 6 (ver. 6.07). The statistical tests used were specified in the figure legends. Statistical significance was defined as *P* < 0.05. The cutoff for a not-significant (ns) P-value to show the exact number is 0.07.

## Electronic supplementary material

Supplementary information
