# Supplementary material for: Mitochondrial retrograde signaling connects respiratory capacity to thermogenic gene expression
Source: Sci Rep. 2017 May 17;7:2013. doi: 10.1038/s41598-017-01879-x (PMC5435730; doi:10.1038/s41598-017-01879-x)
Supplement: Supplementary file 1 — Supplementary information [file 41598_2017_1879_MOESM1_ESM.pdf]

Supplementary information

Mitochondrial retrograde signaling connects respiratory capacity to thermogenic gene expression

Minwoo Nam, Thomas E. Akie, Masato Sanosaka, Siobhan M. Craige, Shashi Kant, John F. Keaney Jr, and Marcus P. Cooper

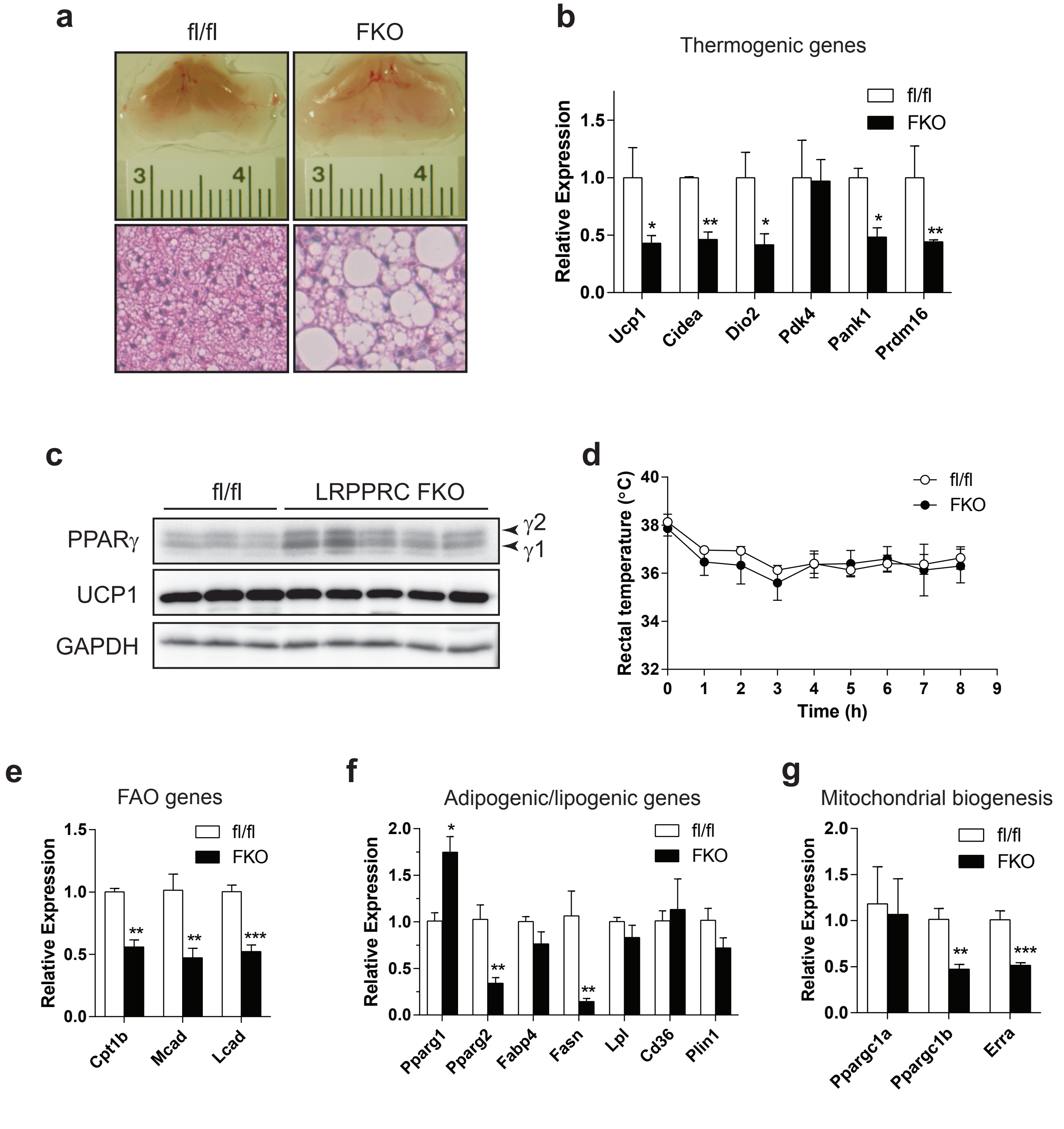

Supplementary Figure 1. Impaired respiratory capacity attenuates thermogenic and oxidaive gene expression in BAT from LRPPRC FKO mice living at 22 °C. (a) Representative images (upper) and H&E staining (lower) of BAT. (b) mRNA levels of thermogenic genes in BAT. (c) Immunoblot of PPARγ, UCP1 and GAPDH (loading control) in BAT. (d) Core temperature of control and LRPPRC FKO mice during acute cold exposure at 4 °C. (e-g) mRNA levels of FAO genes (e), adipogenic/lipogenic gene (f), and mitochondrial biogenesis genes (g) in BAT. (a-d): 9 week-old male mice, n=3-5. Data are mean ± SEM. **P*<0.05, ***P*<0.01, ****P*<0.001, two-tailed unpaired Student's *t*-test (b,e-g).

a

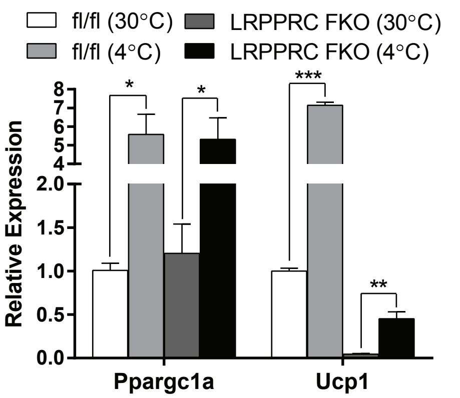

b

		Fold change (30°C vs 4°C)
Ppargc1a	fl/fl	5.539
	FKO	4.415
Ucp1	fl/fl	7.130
	FKO	9.760

c

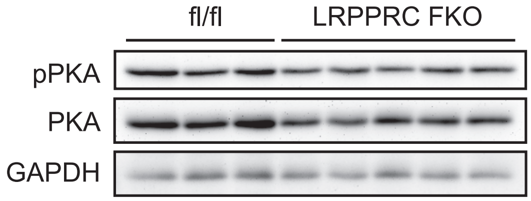

d

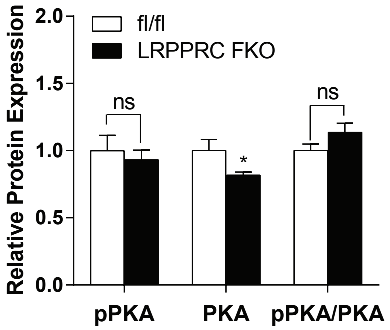

Supplementary Figure 2. β-adrenergic signaling is intact in BAT from LRPPRC FKO mice. (a) mRNA levels of cold-responsive genes in BAT of thermoneutral-acclimated (30 °C) or cold-exposed (4 °C) mice. (b) Table showing fold change for 30 °C vs. 4 °C for the genes from (a). (c) Immunoblot of phosphorylated PKA (pPKA Thr197), total PKA and GAPDH (loading control). (d) Quantification of pPKA and total PKA relative to GAPDH. (a): 12-14 week-old male, n=3-5 (c-d): 12 week-old male mice, n=3-5. Data are mean ± SEM. *P<0.05, **P<0.01, ***P<0.001, two-tailed unpaired Student's t-test (a,d).

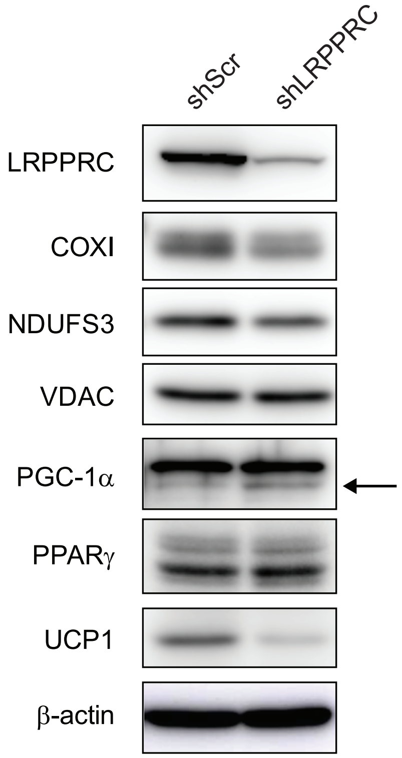

Supplementary Figure 3. Knockdown of LRPPRC in brown adipocytes. Shown is immunoblot of LRPPRC, COXI (mitochondrial-encoded ETC subunit), NDUFS3 (nuclear-encoded ETC subunit), VDAC, PGC-1α, PPARγ, UCP1 and β-actin (loading control).

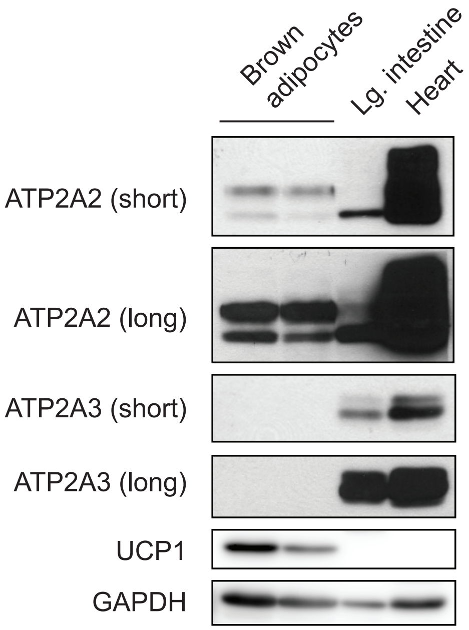

Supplementary Figure 4. ATP2A2 is expressed but ATP2A3 is undetectable in *in vitro*-differentiated brown adipocytes. Shown is immunoblot of ATP2A2, ATP2A3, UCP1 and GAPDH (loading control). short: short exposure, long: long exposure. Mouse heart was used as a postivie control for ATP2A2 and ATP2A3; mouse large (Lg) intestine as a positive control for ATP2A3.

Table S1. Primer used for RT-qPCR

Primer	Forward (5' -> 3')	Reverse (5' -> 3')
Cd36	TCTTCCAGCCAATGCCTTTG	TGGAGATTACTTTTTCAGTGCAGAA
Cidea	TGCTCTTCTGTATCGCCCAGT	GCCGTGTTAAGGAATCTGCTG
Cox5b	GCTGCATCTGTGAAGAGGACAAC	CAGCTTGTAATGGGTTCCACAGT
Cpt1b	TTATTAAGAACACAAATGTGCAAGCA	TTGCGGCGATACATGATCA
Ctsl	TCTGTTGCTATGGACGCAAG	ATAGCCATAGCCCACCAACA
Cycs	GCAAGCATAAGACTGGACCAAA	TTGTTGGCATCTGTGTAAGAGAATC
Dio2	CAGTGTGGTGCACGTCTCCAATC	TGAACCAAAGTTGACCACCAG
Erra (Esrra)	GCAGGGCAGTGGGAAGCTA	CCTCTTGAAGAAGGCTTTGCA
Fabp4	ACAGCTCCTCCTCGAAGGTTT	AAGCCCACTCCCACTTCTTTC
Fasn	GAAAGGACCTGCCCAATCTCT	AAGGACCACACAGCCTCGTAA
Lcad	GGCTGGTTAAGTGATCTCGTGAT	ATGGGCAGGCGATCGA
Lpl	CTCGCTCTCAGATGCCCTAC	CCATCCTCAGTCCCAGAAAA
Lrpprc	GACTTCCTGGCAAAGATGGA	ACCTTCAATGTCCCCAACAT
Mcad	AACACTTACTATGCCTCGATTGCA	CCATAGCCTCCGAAAATCTGAA
Pank1	GTCAGCATCCTGGCAGTGTA	CTAGGAATGTCCCACCTCCA
Pdk4	CCGCTTAGTGAACACTCCTTC	TCTACAAACTCTGACAGGGCTTT
Plin1	CTCTGGGAAGCATCGAGAAG	AAGGGGCTGACTCCTTGTCT
Pparg1	CGGGCTGAGAAGTCACGTTC	GAATATCAGTGGTTCACCGCTTC
Pparg2	GCATGGTGCCTTCGCTGA	TGGCATCTCTGTGTCAACCATG
Ppargc1a	AATGCAGCGGTCTTAGCACT	TTGTGGCTTTTGCTGTTGAC
Ppargc1b	GCCTCTCCAGGCAGGTTCA	TAGAGAACTCAGTCCAGAAGGCTTT
Prdm16	TCTCCGAGATCCGAAACTTCA	GATCTCAGGCCGTTTGTCCAT
Sdha	GCTGGTGTGGATGTCACTAAGG	CCCACCCATGTTGTAATGCA
Ucp1	ACTGCCACACCTCCAGTCATT	CTTTGCCTCACTCAGGATTGG
Uqcrb	CGGGCCGATCTGCTGTT	ACCACTTTCGAAAACCATCCA
Tbp	ACCCTTCACCAATGACTCCTATG	TGACTGCAGCAAATCGCTTGG
36b4	TGCAGATCGGGTACCCAACT	ACGCGCTTGTACCCATTGA
18S	AGTCCCTGCCCTTTGTACACA	CGATCCGAGGGCCTCACTA
ND1	CCCCTTCGACCTGACAGAAG	GGGCCGGCTGCGTATT
ND6	ACAAAGATCACCCAGCTACTACCAT	TTGATGATGTTGGAGTTATGTTGGA
ATP6	AATTACAGGCTTCCGACACAAAC	TGGAATTAGTGAAATTGGAGTTCCT
COXI	TTTTCAGGCTTCACCCTAGATGA	CCTACGAATATGATGGCGAAGTG
CYTB	AGACAACTACATACCAGCTAATCCACTAA	GAATGGCGTATGCAAATAGGAAA

Table S2. Primer used for ChIP assays

Primer	Forward (5' -> 3')	Reverse (5' -> 3')
Cd36	GGCTCAAATCAGTTCCGTTG	TTTCTGGTGAAGACCCATCA
Cpt1b	GCCCTGGAATTAGGGAAAAG	GTGAGCATGGTTGCATCAGT
Fabp4	TTCCCAGCAGGAATCAGGTAG	CTGGGAACTCCATTTGCTCTC
Gapdh TSS	GCTCTCTGCTCCTCCCTGTTC	TGGCAACAATCTCCACTTTGC
Lpl	CGGTAGGCAAACTGGAGTCTA	AAACGGTAACGAGGCTCAAC
Plin	ACCCTCGCCCTTAGGATCT	CCTGAAAGCTCTGCTGACAA
Pank1	ACTTTCGTGGGGCGGGGATG	TCGGGAGGAATGCCGGAGGA
Pdk4	GCAACCAAGTCGTTACAGCGTCC	ACCTCTCTCGTCCTCCCGTTC
Ucp1 enhancer	TCACTCCTCTACAGCGTCACAGA	TCTGGCAGGAAGAGTGGAAAG
